# Analyzing Type 2 Diabetes Associations with the Gut Microbiome in Individuals from Two Ethnic Backgrounds Living in the Same Geographic Area

**DOI:** 10.3390/nu13093289

**Published:** 2021-09-21

**Authors:** Manon Balvers, Mélanie Deschasaux, Bert-Jan van den Born, Koos Zwinderman, Max Nieuwdorp, Evgeni Levin

**Affiliations:** 1 Department of Internal and Vascular Medicine, Amsterdam University Medical Centers, 1105 AZ Amsterdam, The Netherlands; m.balvers@amsterdamumc.nl (M.B.); b.j.vandenborn@amsterdamumc.nl (B.-J.v.d.B.); m.nieuwdorp@amsterdamumc.nl (M.N.); 2 HorAIzon BV, 2625 GZ Delft, The Netherlands; 3 Department of Clinical Epidemiology and Biostatistics, Amsterdam University Medical Centers, 1105 AZ Amsterdam, The Netherlands; m.deschasaux@eren.smbh.univ-paris13.fr (M.D.); a.h.zwinderman@amsterdamumc.nl (K.Z.); 4 Department of Public and Occupational Health, Amsterdam University Medical Centers, 1105 AZ Amsterdam, The Netherlands

**Keywords:** type 2 diabetes, ethnicity, gut microbiome, metformin, treatment-naïve, HELIUS study

## Abstract

It is currently unknown whether associations between gut microbiota composition and type 2 diabetes (T2D) differ according to the ethnic background of individuals. Thus, we studied these associations in participants from two ethnicities characterized by a high T2D prevalence and living in the same geographical area, using the Healthy Life In Urban Settings (HELIUS) study. We included 111 and 128 T2D participants on metformin (Met-T2D), 78 and 49 treatment-naïve T2D (TN-T2D) participants, as well as a 1:1 matched group of healthy controls from, respectively, African Surinamese and South-Asian Surinamese descent. Fecal microbiome profiles were obtained through 16S rRNA gene sequencing. Univariate and machine learning analyses were used to explore the associations between T2D and the composition and function of the gut microbiome in both ethnicities, comparing Met-T2D and TN-T2D participants to their respective healthy control. We found a lower α-diversity for South-Asian Surinamese TN-T2D participants but no significant associations between TN-T2D status and the abundance of bacterial taxa or functional pathways. In African Surinamese participants, we did not find any association between TN-T2D status and the gut microbiome. With respect to Met-T2D participants, we identified several bacterial taxa and functional pathways with a significantly altered abundance in both ethnicities. More alterations were observed in South-Asian Surinamese. Some altered taxa and pathways observed in both ethnicities were previously related to metformin use. This included a strong negative association between the abundance of *Romboutsia* and Met-T2D status. Other bacterial taxa were consistent with previous observations in T2D, including reduced butyrate producers such as *Anaerostipes hadrus*. Hence, our results highlighted both shared and unique gut microbial biomarkers of Met-T2D in individuals from different ethnicities but living in the same geographical area. Future research using higher-resolution shotgun sequencing is needed to clarify the role of ethnicity in the association between T2D and gut microbiota composition.

## 1. Introduction

Type 2 Diabetes (T2D) is considered as a major global public health concern [1]. Differences in prevalence across different ethnicities are known, both between countries as well as between ethnicities living in the same geographical area [2,3]. In the latter, the prevalence of T2D is often higher in ethnic minority groups compared to the host population, as depicted in several cohort studies [2,3,4,5]. In the Healthy Life In Urban Setting (HELIUS) study, South-Asian Surinamese in Amsterdam had the highest prevalence of T2D among all ethnic groups, and African Surinamese also showed a much higher prevalence compared to the Dutch population [3]. However, the underlying mechanism of this difference remains unclear [2]. In this regard, T2D is often caused by a combination of multiple genetic and environmental factors, including lifestyle and biological factors, which may also partially differ between ethnicities [1,6]. Recently, the gut microbiome, composed of trillions of bacteria, viruses and fungi and their corresponding genes, was also suggested to play a role in the development of T2D [7,8,9,10,11].

In addition to its known role in several fundamental processes in the host, including nutrient metabolism and the host immune response, the gut microbiome is indeed also proposed to be involved in host glycemic control and associated with insulin resistance [10,11,12,13,14,15]. Several studies have shown differences in microbiome structure or function between healthy and (pre)diabetic subjects [7,8,9,10,16,17,18,19,20,21]. A lower abundance of butyrate producing bacteria in (pre)diabetic subjects was identified in multiple studies, but other findings were inconsistent [7,9,10,19,20]. Recently, oral antidiabetic drugs, and especially metformin, were shown to alter the gut microbiome composition and function, which might have confounded some of the previous results [9,22,23,24]. Furthermore, across those studies, individuals with different ethnic origins were included, which could explain some of the inconsistencies between studies. Some studies even showed that some associations could not be replicated in another ethnic cohort within the same study [8,9,21,25]. This might suggest that the identified associations could be ethnic specific, in line with generally observed differences in microbiome composition across different ethnicities [21,26,27,28]. However, the influence of potential confounders, such as different study designs, sample size, geographical location or medication use, on the observed differences could not be excluded in these previous T2D studies.

From the above observations, the question arises if different gut microbiome profiles in T2D are indeed observed in subjects from different ethnic descent. Insights herein could improve future applications of the gut microbiome in personalized prevention, diagnostic tools or therapeutic strategies. Therefore, in the ongoing HELIUS study, we analyzed the differences in gut microbiome composition and function between healthy and T2D subjects of South-Asian Surinamese and African Surinamese descent, two ethnic minority groups with a high prevalence of T2D living in Amsterdam (NL), taking into account their metformin medication status. 

## 2. Materials and Methods

### 2.1. Study Population

The HELIUS study is an ongoing study, at baseline including 18–70 years old residents of Amsterdam, the Netherlands. Participants were randomly recruited from the municipal registry, stratified by their ethnic origin, being of either Dutch, South-Asian Surinamese, African Surinamese, Turkish, Moroccan or Ghanaian descent (mainly first-generation immigrants). A detailed description of the study design, study population and rationale is provided elsewhere [29,30]. In the current analyses, we included only participants who provided a stool sample during the baseline visit between 2011 and 2015 and for whom gut microbiome profiles were available. The HELIUS study was approved by the Academic Medical Center (AMC) Medical Ethics Committee, and all participants provided informed consent.

### 2.2. Baseline Data Collection

Recruited participants completed a questionnaire including items related to sociodemographic characteristics, lifestyle (including physical activity, smoking, alcohol use and dietary habits) and health status. They underwent a physical examination, including measurements of anthropometric characteristics (e.g., height, weight and waist circumference) and blood pressure, and a review of current medication. Overnight fasted blood samples were drawn and used for determination of HbA1c, glucose and lipid profile. Furthermore, a subset of participants collected their own stool sample at home using a collection tube. They were asked to bring the sample to the research location within 6 h after collection or otherwise to store it in the freezer overnight and bring it the next day. At the research location, samples were temporarily stored at −20 °C, before daily transport to the AMC, where they were checked for conformity and stored at −80 °C. Information regarding participants’ antibiotic use in the past three months, probiotic use and whether they had diarrhea in the past week was recorded when participants handed in the sample. A subsample of the HELIUS cohort also provided detailed dietary intake data from ethnic-specific food frequency questionnaires (FFQs), as previously described [31,32].

### 2.3. Profiling of Fecal Microbiota Composition

Sequencing of the stool samples was performed at the Wallenberg Laboratory (Sahlgrenska University of Gothenburg, Gothenburg, Sweden). Total genomic DNA was extracted from a 150 mg fecal sample aliquot using a repeated bead beating method [33]. Fecal microbiome composition was profiled by sequencing the V4 region of the 16S rRNA gene on an Illumina MiSeq instrument (Illumina RTA v1.17.28; MCS v2.5, San Diego, CA, USA) with 515F and 806R primers designed for dual indexing [34] and the V2 Illumina kit (2 × 250 bp paired-end reads). Here, 16S rRNA genes from each sample were amplified in duplicate reactions in volumes of 25 μL, which contained 1× Five Prime Hot Master Mix (5PRIME GmbH, Hamburg, Germany), 0.4 mg mL^−1^ BSA, 5% dimethylsulfoxide, 200 nM of each primer and 20 ng of genomic DNA. PCR was carried out under the following conditions: initial denaturation at 94 °C for 47 min; followed by 25 cycles of denaturation at 94 °C for 45 s; annealing at 52 °C for 60 s; elongation at 72 °C for 90 s; and a final elongation step at 72 °C for 10 min. Duplicates were combined, purified with the PCR Clean-Up kit (Macherey-Nagel, Düren, Germany) and the NucleoSpin Gel, and quantified using the Quant-iT PicoGreen dsDNA kit (Invitrogen, Waltham, MA, USA). Purified PCR products were diluted to 10 ng/μL and pooled in equal amounts. To remove short amplification products, these pooled amplicons were purified again using Ampure magnetic purification beads (Agencourt, Beverly, MA, USA). Negative controls were included for each sample. Gel electrophoresis was used to confirm the absence of detectable PCR products in these negative controls. Positive controls were not included in these runs. However, the protocol used to analyze the samples was optimized using mock samples. Libraries for sequencing were prepared by mixing the pooled amplicons with PhiX control DNA purchased from Illumina. This input DNA contained 15% PhiX and had a concentration of 3 pM and resulted in the generation of about 700 K clusters/mm^2^ and an overall percentage of bases with quality score higher than 30 (Q30) higher than 70%. All analytical procedures were blinded for ethnicity (but not randomized).

### 2.4. Bioinformatics Pipeline

USEARCH (v11.0.667_i86linux64, [35]) was used to process the raw sequencing reads. To merge the paired-end reads, the merging step (using ‘fast_mergepairs’ command) was performed with a maximum of 30 allowed differences in the overlapping regions (‘maxdiffs’) and a maximum of 1 expected error (‘fastq_maxee’) as a quality filter threshold (using the ‘fastq_filter’ command). After merging the paired-end reads and quality filtering, Amplicon Sequence Variants (ASVs) were obtained by dereplicating the remaining contigs and denoising the unique sequences using the UNOISE3 algorithm. Subsequently, all merged reads were mapped against the resulting ASVs to produce an ASV table. ASV sequences longer than 260 base pair or shorter than 250 base pair (i.e., ASVs not matching the expected amplicon length) were filtered out. Taxonomy was assigned using the ‘assignTaxonomy’ function from the ‘dada2′ R package (v1.12.1) and the SILVA reference database (v.132) [36,37]. MAFFT (v. 7.427 [38,39]) using default settings was then used to align the ASV sequences. From the resulting multiple sequence alignment, a phylogenetic tree was constructed with the ‘double precision’ build of FastTree (v. 2.1.11 [40]) using a generalized time-reversible model (‘-gtr’). The tree, taxonomy and ASV table were integrated using the ‘phyloseq’ R package (v. 1.28.0 [41]). Lastly, the ‘vegan’ R package (v 2.5-6 [42]) was used to rarefy the ASV table to 14,932 counts per sample. Of all 6056 sequenced samples, samples with insufficient counts (<5000 counts per sample, *n* = 24) were excluded at this rarefaction stage, resulting in a final complete dataset of 6032 samples with 22,532 ASVs. Lastly, this rarefied ASV table was inputted to PICRUSt2 (v 2.2.0_b) with default parameters to predict MetaCyc pathway abundances per sample [43].

### 2.5. Ascertainment of T2D and Controls

Type 2 diabetes was defined based on either fasting glucose levels ≥7.0 mmol/L, fasting HbA1c levels ≥48 mmol/mol or use of antidiabetic medication. To account for the known confounding effect of metformin on the gut microbiome [9,22,23,24], T2D cases were further divided into ‘T2D cases on metformin treatment’ (Met-T2D) and ‘T2D cases not taking any antidiabetic medication’ (treatment-naïve, TN-T2D). T2D cases not classified as Met-T2D or TN-T2D were excluded. 

Healthy controls were included if they were normoglycemic (both fasting glucose levels <5.6 mmol/L and fasting HbA1c levels <39 mmol/mol), did not self-report diabetes and did not fulfil more than 1 of the following metabolic syndrome criteria: high blood pressure (systolic blood pressure ≥130 mmHg or diastolic blood pressure ≥85 mmHg or use of blood pressure lowering medication), high blood triglycerides (≥1.7 mmol/L or lipid lowering medication), reduced HDL cholesterol (<1.03 mmol/L (male), <1.29 mmol/L (female) or lipid lowering medication) or high fasting plasma glucose (≥5.6 mmol/L or glucose lowering medication). The central obesity component (waist circumference ≥80 cm (female), ≥90 cm (Hindu male) and ≥94 (non-Hindu male)) was not taken into account due to its high prevalence. In case of missing data for a particular criterion, this criterion was considered as fulfilled. 

Participants were excluded if they had taken antibiotics in the past three months or if this variable was unknown. In addition, healthy controls were also excluded in case they had missing values for at least one of the following values: fasting glucose levels, fasting HbA1c levels, antidiabetic medication use, metabolic syndrome status, age, sex or BMI.

Based on low number of T2D cases in the Ghanaian, Turkish, Moroccan and Dutch groups, the present study only focused on the African Surinamese (Met-T2D: *n* = 111, TN-T2D: *n* = 78) and South-Asian Surinamese (Met-T2D: *n* = 128, TN-T2D: *n* = 49). Healthy controls from the same ethnic group were selected for Met-T2D and TN-T2D cases using a 1:1 propensity score matching based on age, sex and BMI. (MatchIt R package v. 3.0.2. [44]). The matching was performed separately by T2D treatment status, so that the same healthy participant could serve as control for both the Met-T2D and TN-T2D cases.

### 2.6. Statistical Analysis

Since T2D cases were matched to controls based on only a limited number of general variables [45], and we did not observe a positively skewed distribution of within pairs correlations for bacterial features. We performed unpaired analyses for all downstream analyses. 

Anthropometric and clinical values are summarized with mean ± standard deviation or median (Inter Quartile Range) for normally and non-normally distributed variables, respectively. Differences in these variables between controls and diabetes subjects were compared with two sample *t*-tests or Wilcoxon rank-sum tests, respectively. Counts (%) are provided for categorical variables and compared with a Fisher exact test or Chi-squared test.

We obtained dietary patterns scores for a subset of individuals by performing a principal components analysis with VARIMAX rotation (function ‘principal’ from psych R package v. 2.0.9 [46]) on 44 nonzero food group variables from the ethnic-specific FFQs. Based on the interpretability of the patterns and the scree plot, 2 patterns were retained. Food items with an absolute factor loading of ≥0.3 were considered to contribute significantly to a diet pattern. A score for each diet pattern, representing the adherence to this pattern, for each individual was used in the sensitivity analyses.

The diversity of gut microbiota for each individual was assessed with several α-diversity indices calculated at the ASV level, namely richness (number of unique ASVs, function ‘specnumber’ from vegan R package 2.5-6 [42]), Faith’s PD (function ‘pd’ from picante R package v.1.8.2. [47]), Shannon index and inverse Simpson index (both with function ‘diversity’ from vegan R package [42]). Linear regression, with each α-diversity index as outcome variable, was performed to test the effect of T2D on the α-diversity index. Models were tested without adjustment and with adjustment for age, sex and BMI (“covariate-adjusted” model) and additionally for proton pump inhibitor (PPI) use, statin use, beta blocker use and use of medication working on the renin-angiotensin system (“covariate-and-medication-adjusted” model). For participants with available dietary information from FFQs, the same models were tested as well as models with an additional adjustment for dietary scores (“diet-adjusted” model, “covariate-and-diet-adjusted” models and “covariate-medication-and-diet-adjusted” models).

The interindividual dissimilarities in gut microbiota composition (β-diversity) were assessed with both the unweighted and weighted UniFrac distances (function ‘UniFrac’ from phyloseq v 1.30.0 R package [48]) and the Bray–Curtis dissimilarity index (function ‘vegdist’ from vegan R package [42]) all obtained at the ASV level. Dissimilarities were plotted using Principal Coordinate Analysis (PCoA, function ‘pcoa’ of ape v5.3 R package [49]) on the first 2 components. Statistical differences in β-diversity estimates between T2D cases and healthy controls were tested using PERMANOVA (function ‘adonis’ from vegan R package [42], 10,000 permutations). Unadjusted, ”covariate-adjusted” and “covariate-and-medication-adjusted” models were used in both the full dataset and in the subset of participants with available dietary scores. In the latter the “diet-adjusted”, “covariate-and-diet-adjusted” and “covariate-medication-and-diet-adjusted” models were also tested. 

All of the above-described analyses regarding α- and β-diversity were performed for each ethnicity separately and stratified by T2D status (Met-T2D vs. their matched healthy controls or TN-T2D vs. their matched healthy controls). Interactions between T2D status and ethnicity were tested by introducing the product of T2D status and ethnicity in a model already including T2D status and ethnicity. 

Within each ethnicity, read counts of individual ASVs were compared between T2D cases (Met-T2D or TN-T2D, separate analysis) and their controls to identify differentially abundant ASVs. Only ASVs with on average ≥ 3 reads per sample (≥0.02%) in the specific dataset under consideration were included in the analysis. Since ASV abundance is not normally distributed and no golden standard for ASV analysis is available, we applied a sensitivity analysis with different models and transformation methods (data not shown) to select significant differentially abundant ASVs. While we observed that results were dependent on the chosen model or transformation in some cases, we decided to use the Wilcoxon rank-sum test on the untransformed read counts to compare the abundances, since this model uses the least assumptions about the distribution. *p*-values were corrected for multiple comparisons using Benjamini–Hochberg correction (*p*. adjust) [50]).

For each T2D status, ASVs that remained significant after this correction in either of the two ethnicities were further analyzed with a sensitivity analysis to disentangle the effect of potential confounders. Therefore, linear regression models with arcsin square-root transformed relative abundances of each ASV as output were run for each ethnicity separately, using the same type of adjustments as for the α- and β-diversity measures (unadjusted, “covariate-adjusted”, “covariate-and-medication-adjusted” models in the full cohort and the subset of participants with available diet information; “diet-adjusted”, “covariate-diet-adjusted” and “covariate-medication-and-diet-adjusted” models only in the subset of participants with available diet information). To verify if T2D had a different effect on the abundance of the identified ASVs for a specific ethnicity, we tested the interaction effect between T2D and ethnicity on the abundance of the selected ASVs by introducing the product of T2D status and ethnicity in a model already including T2D status and ethnicity.

To compare the predicted functional differences of the gut microbiome between T2D cases and controls, PICRUSt2 read counts were normalized to relative abundances using total sum scaling. Only pathways with an average abundance ≥ 0.02% in the specific dataset under consideration were included in the analysis. We conducted the same analysis as those conducted for ASV. However, in the sensitivity analysis, pathway abundances were log-transformed after addition of a pseudocount (0.01%).

For all statistical analysis, R v.3.6.2 [51] was used in RStudio (v 1.2.5033). *p*-values ≤ 0.05 (either Benjamini–Hochberg corrected or for terms in (un)adjusted linear models) were considered to be statistically significant for single terms, whereas for interaction terms a criterion of *p*-value ≤ 0.20 was used. Since in the sensitivity analysis of the ASV and pathway abundances *p*-values were no longer corrected for multiple comparisons, ASVs and pathways were only considered as significantly associated with T2D in a specific ethnicity if they were significant in both the Benjamini–Hochberg corrected Wilcoxon analysis and the linear regression models. 

### 2.7. Machine Learning

We built machine learning models to predict T2D status (T2D vs. matched controls) based on microbiome profile for both Met-T2D and TN-T2D status for each ethnicity separately. Models were built on the same features included in the Wilcoxon analyses described above. All models were constructed with the same stability selection procedure to ensure robustness of the results and prevent overfitting. [52] For this, we reshuffled the order of the samples in the original dataset 50 times. After each shuffle, the dataset was split into a training (80% of the data) and a test set (20% of the data) with an equal balance between T2D cases and controls in both sets. The test set was not seen by the model during training. We preprocessed the training dataset by adding a random variable (to enable discrimination of predictive features later) and scaling the dataset with zero mean, unit variance. We then fitted an extremely randomized tree model [53] on this training dataset after tuning the hyperparameters of the model with 5-fold cross validation. Once the model was built, we used the test set to obtain performance measures. Model performance was represented by the Area Under the Curve (AUC). Feature importance was assessed by both the permutation feature importance [54] and Shapley Additive exPlanations (SHAP) importance (function ‘TreeExplainer’ with feature_perturbation = ’tree_path_dependent’ from shap v. 0.35.0 Python package [55]). 

Final model performance, SHAP feature importance and permutation feature importance were obtained by averaging the scores across the different shuffles. Features were selected as important to distinguish T2D cases and healthy controls if they were both present in the top 20 of the average SHAP importance and in the top 20 of the average permutation importance. The machine learning pipeline was implemented in Python v3.7.6, using the scikit-learn (v 0.22.1) package.

## 3. Results

In total, 189 African Surinamese T2D cases (111 Met-T2D and 78 TN-T2D) and 177 South-Asian Surinamese T2D cases (128 Met-T2D and 49 TN-T2D) were included. After matching, 130 African Surinamese and 128 South-Asian Surinamese were selected as healthy controls, some of them being matched to both a Met-T2D and a TN-T2D case. Since both metformin use and ethnicity are known confounders for the gut microbiome [9,22,23,24,26], all analyses were performed according to metformin use and ethnicity (i.e., African Surinamese Met-T2D cases vs. matched controls, African Surinamese TN-T2D cases vs. matched controls, South-Asian Surinamese Met-T2D cases vs. matched controls, and South-Asian Surinamese TN-T2D cases vs. matched controls). Characteristics of participants according to T2D status are provided in Table 1. Despite the propensity-score matching, T2D cases were in general older or had a higher BMI. These differences were particularly observed between South-Asian Surinamese Met-T2D cases and controls, where T2D cases were also more often male. Furthermore, T2D cases had a higher prevalence of all metabolic syndrome components, except for central obesity in South-Asian Surinamese TN-T2D. Regarding medication use, statins were only taken by T2D cases, use of beta blockers and medications targeting the renin-angiotensin system was higher in Met-T2D cases in both ethnic groups, while South-Asian Surinamese Met-T2D cases took more often PPIs. Detailed FFQs diet information was available for about 30% of the subjects and used to generate adherence scores for two dietary patterns, namely adherence to a “healthy” diet (PC1, with significant factor loadings including vegetables, fruit, olive oil and fish) and adherence to a “Western” diet (PC2, with significant factor loadings including fast food, sweets and snacks); see Supplementary Table S1.

### 3.1. Gut Microbiome Diversity Analysis

**Figure 1:**
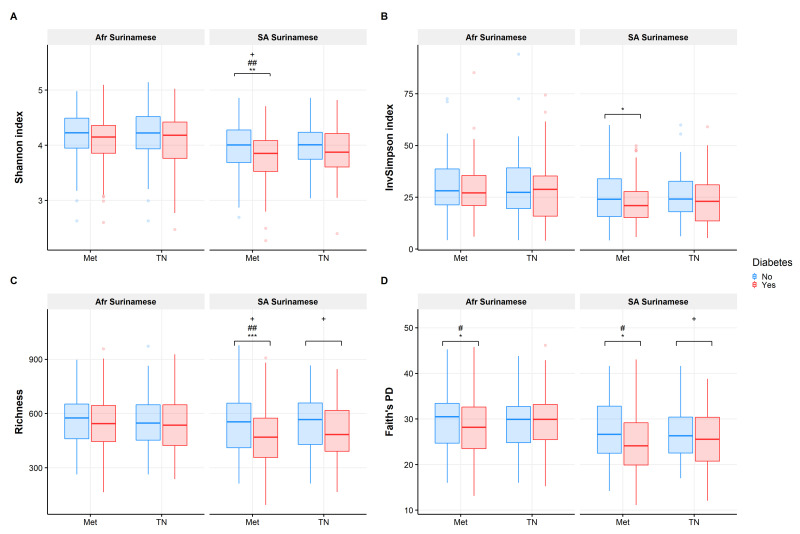
Overview of α-diversity measures in African and South-Asian Surinamese for the Met-T2D (Met) and TN-T2D (TN) datasets (T2D cases and matched controls) for (**A**) Shannon Index, (**B**) inverse Simpson index, (**C**) richness and (**D**) Faith’s PD. Significant differences are indicated with different symbols for different models: * (no adjustment for confounders), # (adjustment for age, sex and BMI) and + (adjustment for age, sex, BMI and medication use) and different number of symbols for different significant levels (*/#/+ = *p* ≤ 0.05, **/##/++ = *p* ≤ 0.01 and ***/###/+++ = *p* ≤ 0.001).

Analyses of α-diversity measures are displayed in Figure 1 and Supplementary Table S2. The diversity of the gut microbiota in South-Asian Surinamese Met-T2D cases was lower compared to their healthy controls, indicated by a lower richness (*p* = 0.000716), Shannon index (*p* = 0.0027), inverse Simpson index (*p* = 0.0169) and phylogenetic diversity (Faith’s PD, *p* = 0.0103). After adjusting for age, sex, BMI and medication use, both South-Asian Surinamese Met-T2D and TN-T2D cases were characterized by a significantly lower richness and diversity (Met-T2D: Shannon, TN-T2D: Faith’s PD) compared to their healthy controls. In African Surinamese, we only observed a significantly lower phylogenetic diversity in Met-T2D cases (*p* = 0.0324) compared to their controls, which remained significant after adjusting for age, sex and BMI. In addition, a significant interaction effect between Met-T2D and ethnicity was observed for the Shannon index and richness, supporting the difference in observed associations between ethnic groups. In the subsample with available dietary information, T2D status was not associated with α-diversity in any of the models (with or without additional adjustment for dietary patterns (see Supplementary Table S3).

**Figure 2:**
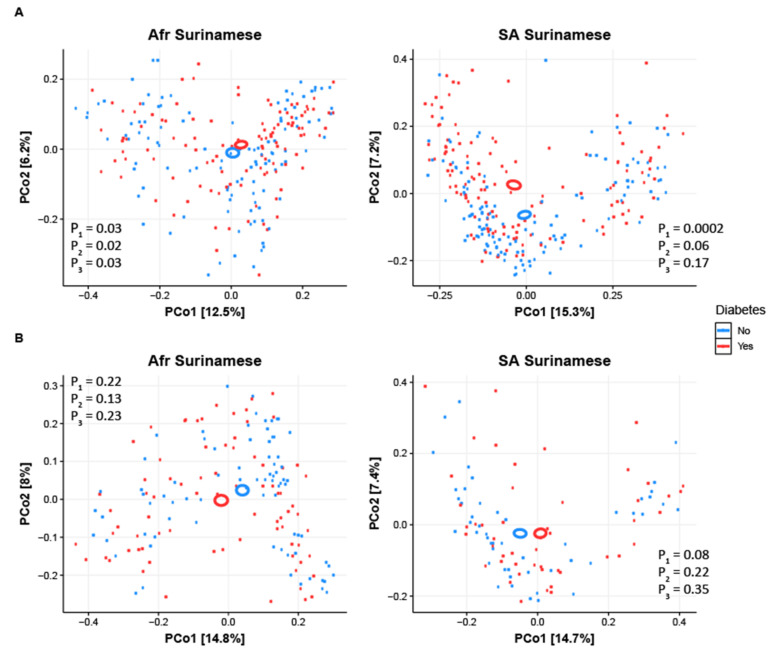
Visualization of the PCoA analysis on the Bray–Curtis dissimilarity index for both African and South-Asian Surinamese in the (**A**) Met-T2D dataset (Met-T2D cases and matched controls) and (**B**) TN-T2D dataset (TN-T2D cases and matched controls) and with *p* values for the (1) PERMANOVA without adjustment, (2) PERMANOVA with adjustment for age, sex and BMI and (3) PERMANOVA with adjustment for age, sex, BMI and medication use.

Although no clear separation was seen visualizing the results of the PCoA using the three β-diversity measures (see Figure 2 for the Bray–Curtis dissimilarity plot), subsequent PERMANOVA analysis (see Supplementary Table S4) revealed dissimilar microbiota compositions between Met-T2D cases and their healthy controls in both ethnicities based on Bray–Curtis (African Surinamese: *p* = 0.0332, South-Asian Surinamese: *p* = 0.0002) and unweighted UniFrac (African Surinamese: *p* = 0.0186, South-Asian Surinamese: *p* = 0.0005). Only South-Asian Surinamese Met-T2D cases had a significantly different weighted UniFrac dissimilarity compared to their healthy controls (*p* = 0.0013), supported by a significant interaction effect between Met-T2D and ethnicity. However, only the effect of Met-T2D on Bray–Curtis dissimilarity in the African Surinamese remained significant after adjusting for age, sex, BMI and medication use. The same analyses in the diet subsample revealed either no significant effects of T2D or the effect vanished after adjusting for the dietary patterns (see Supplementary Table S5).

### 3.2. Analysis of Biomarkers for T2D

**Figure 3:**
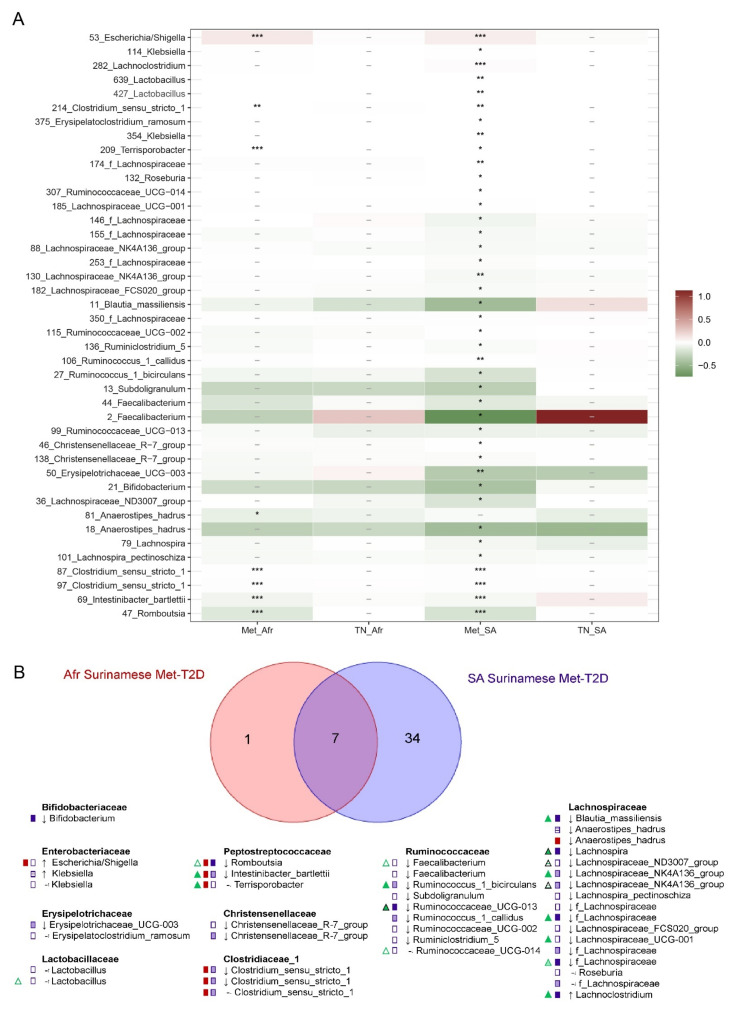
Overview of the Wilcoxon analysis and sensitivity analysis on ASVs for all datasets (T2D cases and their matched controls). (**A**) Overview of the Wilcoxon analysis on the ASVs for all datasets. Only features that were significant in at least one dataset are displayed. Features are displayed as (ASV nr)_(taxonomy name). Significant features after correction for multiple testing are indicated with a *. * *p* ≤ 0.05, ** *p* ≤ 0.01, *** *p* ≤ 0.001, not significant features are indicated with a -; no symbol indicates that the feature was filtered out before the analysis was applied. Color of each feature indicates the difference in median relative abundance (in %) for this ASV. Green indicates a lower median relative abundance of the feature in T2D cases compared to their healthy controls, red indicates a higher median relative abundance of the feature in T2D cases compared to their healthy controls and white indicates an equal median relative abundance of the feature in both T2D cases and their healthy controls. Met_Afr = Met-T2D dataset for African Surinamese, TN_Afr = TN-T2D dataset for African Surinamese, Met_SA = Met-T2D dataset for South-Asian Surinamese and TN_SA = TN-T2D dataset for South-Asian Surinamese. (**B**) Overview of the sensitivity analysis for all significant ASVs identified with the multiple testing corrected Wilcoxon analysis on the Met-T2D datasets. ASVs are indicated by taxonomy name. Duplicate taxonomy names indicate different ASVs. The color of the rectangle in front of the ASVs indicates the ethnicity for which the model was performed. Empty rectangle indicates the ASV was significant in the Wilcoxon test after adjusting for multiple testing, a partly transparent box indicates T2D was significant after adjusting for age, sex and BMI, using linear models with arcsin-root transformed ASV abundance as dependent variable, whereas a fully filled box indicates T2D was significant after adjusting for age, sex, BMI and use of PPI, statins, beta blockers or drugs targeting the renin-angiotensin system. Rectangles filled with stripes indicates that T2D was significant in the model adjusted for age, sex, BMI and medication use, but not in the model only adjusted for age, sex and BMI. Results for adjusted models are only shown if the ASV was significantly different for that ethnicity in the multiple testing corrected Wilcoxon test. Triangles indicates the results of the interaction effect between T2D and ethnicity. Filling patterns for triangles are equivalent to the filling patterns for the rectangles, with an addition of a black circumference indicating the interaction term was not significant in the unadjusted model.–equal median relative abundance of ASV between Met-T2D cases and healthy controls, ↓ lower median relative abundance of ASV and/or negative regression coefficient in Met-T2D cases compared to healthy controls, ↑ higher median relative abundance of ASV and/or positive regression coefficient in Met-T2D cases compared to healthy controls.

To identify differentially abundant ASVs between T2D cases and matched healthy controls, we applied univariate analyses on each dataset on a prefiltered subset of ASVs (see Supplementary Table S6). After adjusting the results of the Wilcoxon analysis for multiple testing, 41 ASVs were significantly different between South-Asian Surinamese Met-T2D cases and their healthy controls. Fewer significant ASVs were identified in the African Surinamese, where only eight ASVs were significantly different between Met-T2D cases and their healthy controls (see Figure 3A). In both ethnicities, the identified ASVs were more often less abundant in Met-T2D cases. Whereas in both ethnicities TN-T2D was associated with the abundance of some ASVs based on the unadjusted *p*-values, remarkably none of them remained significant after adjusting for multiple testing.

Several ASVs assigned to the *Peptostreptococcaceae* family (*Romboutsia*, *Intestinibacter bartlettii* and *Terrisporobacter*), several ASVs assigned to *Clostridium sensu stricto 1*, and one ASV assigned to *Escherichia/Shigella* were associated with Met-T2D in both ethnicities (see Figure 3A), with a lower abundance of the *Peptostreptocacceae* and *Clostridium* and a higher abundance of *Escherichia/Shigella* ASVs in Met-T2D subjects compared to their matched controls. By contrast, some ASVs were uniquely identified in either African Surinamese or South-Asian Surinamese. For example, one ASV assigned to *Anaerostipes hadrus* (ASV 81) was only identified in African Surinamese, while several ASVs assigned to members of the *Lachnospiraceae*, *Lactobacillaceae*, *Christensenellaceae*, *Erysipelotrichaceae* and *Ruminococcaceae* families were unique for the South-Asian Surinamese.

To further disentangle the effect of age, sex, BMI and medication use on our results, we performed a sensitivity analysis using multiadjusted linear regression models (with arcsin-root-transformed ASV abundance) for all ASVs that were significantly associated with Met-T2D in at least one ethnicity after multiple testing correction in the previous analyses (see Figure 3B). For robustness, associations between ASVs and Met-T2D were only considered significant if they were significant in both the Wilcoxon test and sensitivity analysis. Interactions between Met-T2D and ethnicity were tested to verify potential differential associations between Met-T2D status and ASVs according to ethnicity. We observed that for the African Surinamese the effect of Met-T2D remained significant for all selected ASVs, whereas this was not the case for the South-Asian Surinamese (Supplementary Table S7). In South-Asian Surinamese, the effect of Met-T2D remained significant in only eight of the ASVs (mainly assigned to the *Lachnospiraceae* family) in both “covariate-adjusted” models and “covariate-and-medication-adjusted” models. For most of these ASVs, the Met-T2D effect was uniquely significant for the South-Asian Surinamese, with significant interactions between Met-T2D status and ethnicity. In the subgroups with available dietary information, African Surinamese Met-T2D cases still showed a significant lower abundance for several ASVs belonging to the *Peptostreptococcacceae* family and *Clostridium sensu stricto 1* after adjusting for age, sex, BMI and dietary patterns compared to their controls, and for *Romboutsia* also after adjusting for medication use. In South-Asian Surinamese, Met-T2D cases had a lower abundance of *Bifidobacterium* compared to their controls after adjusting for age, sex, BMI, medication use and dietary patterns, whereas the abundance of *Romboutsia* and *Clostridium sensu stricto 1* was only significantly lower after adjusting for age, sex, BMI and dietary patterns (see Supplementary Table S7).

**Figure 4:**
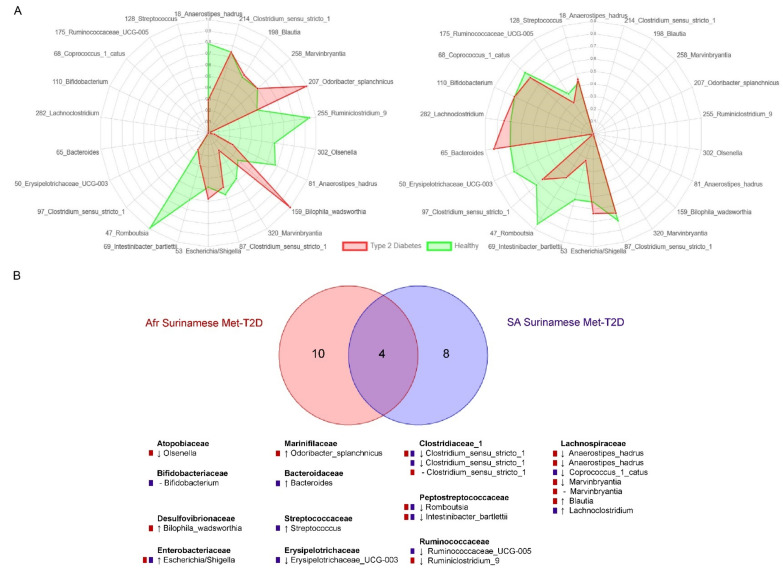
Overview of identified biomarkers in machine learning models for the African Surinamese Met-T2D dataset (Met-T2D cases and matched controls) and the South-Asian Surinamese Met-T2D dataset (Met-T2D cases and matched controls). (**A**): Abundance of identified biomarkers for the African Surinamese Met-T2D dataset (left) and the South-Asian Surinamese Met-T2D dataset (right). Biomarkers are indicated with (ASV nr)_(taxonomy). Values are scaled medians of zero mean unit variance scaled features. Only the features which were selected for the ethnic group under consideration are displayed. (**B**) Overlap in selected ASVs for classifying Met-T2D cases in the machine learning analysis across ethnicities. ASVs are indicated by taxonomy name. Duplicate taxonomy names indicate different ASVs. Color of box in front of the ASVs indicates the ethnicity for which the ASV was important. –equal median relative abundance of ASV between Met-T2D cases and matched healthy controls, ↓ lower median relative abundance of ASV in Met-T2D cases compared to matched healthy controls, ↑ higher median relative abundance of ASV in Met-T2D cases compared to matched healthy controls.

To assess if the gut microbiome composition could distinguish T2D cases from controls, which would be useful for future diagnostic applications, we trained extremely randomized tree classifiers on the ASV read counts. For both ethnicities, it was not possible to separate TN-T2D cases from their matched controls with high accuracy, indicated by an average AUC of 0.64 ± 0.09 and 0.46 ± 0.09 for the South-Asian Surinamese and African Surinamese, respectively. By contrast, Met-T2D status could be predicted with a high accuracy in both ethnicities, as shown by an average AUC of 0.81 ± 0.05 and 0.78 ± 0.06 for South-Asian Surinamese and African Surinamese, respectively. The most important features separating Met-T2D cases from their matched controls for each ethnicity were then obtained by combining two feature importance scores [54,55], resulting in 14 biomarkers for the African Surinamese (Figure 4A, left) and 12 biomarkers for the South-Asian Surinamese (Figure 4A, right). Of these, six and seven markers, respectively, were also identified as associated with Met-T2D status in the Wilcoxon analysis and in some cases even in the sensitivity analysis. ASVs assigned to *Escherichia/Shigella*, *Clostridium sensu stricto 1*, *Romboutsia* and *Intestinibacter bartlettii* were important for classifying Met-T2D cases in both ethnicities (Figure 4B) of which *Romboutsia* was the most important feature in both ethnicities and with both feature importance scores. In addition, for African Surinamese, several *Lachospiraceae*, including *Anaerostipes hadrus*, were among the most important features as well as ASVs assigned to *Olsenella* and *Bilophila wadsworthia*. For South-Asian Surinamese other *Lachnospiraceae*, including *Lachnoclostridium*, as well as ASVs assigned to *Bifidobacterium*, *Streptococcus* and *Erysipelotrichaceae_UCG-003* were selected.

### 3.3. Functional Analysis

**Figure 5:**
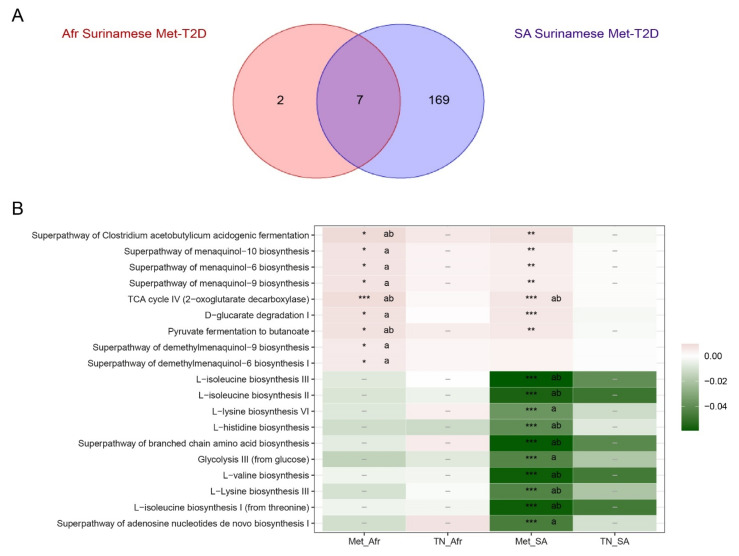
Overview of significant pathways in the Wilcoxon analysis. (**A**) Overlap in significant pathways with the Wilcoxon analysis after correction for multiple testing, without adjusting for confounders, in African Surinamese and South-Asian Surinamese in the Met-T2D datasets (Met-T2D cases and matched controls). (**B**) Overview of the most significant pathways selected by Wilcoxon analysis according to T2D status in both ethnicities. Significant pathways after correction for multiple comparison are indicated with a *. * *p* ≤ 0.05, ** *p* ≤ 0.01, *** *p* ≤ 0.001, not significant pathways are indicated with a -, no symbol indicates the pathway was filtered out before the analysis was applied. Color of each pathway indicates the difference in median relative abundance (in %) for this pathway. Green indicates a lower median relative abundance of the pathway in T2D cases compared to their matched healthy controls, red indicates a higher median relative abundance of the pathway in T2D cases compared to their matched healthy controls and white indicates an equal median relative abundance of the pathway in both T2D cases and their matched healthy controls. Only significant pathways were tested with a linear regression model, using log-transformed relative abundances of the pathways as dependent variable, to adjust for (a) age, sex and BMI or (b) age, sex, BMI and use of PPI, statins, beta blockers or medication working on the renin-angiotensin system. Significant pathways after adjustment are indicated with the corresponding character. Met_Afr = Met-T2D dataset (Met-T2D cases and matched controls) for African Surinamese, TN_Afr = TN-T2D dataset (TN-T2D cases and matched controls) for African Surinamese, Met_SA = Met-T2D dataset (Met-T2D cases and matched controls) for South-Asian Surinamese and TN_SA = TN-T2D dataset (TN-T2D cases and matched controls) for South-Asian Surinamese.

Lastly, we analyzed the difference in functional potential of the gut microbiome between T2D cases and their healthy controls in all datasets. Univariate analyses on a prefiltered set of MetaCyc pathway abundances predicted by PICRUSt2 revealed 176 significantly altered pathways in South-Asian Surinamese Met-T2D cases compared to their matched controls. However, in African Surinamese, only nine pathways were significantly altered in Met-T2D cases (see Figure 5A and Supplementary Table S8). The abundances of these pathways identified in the African Surinamese were all higher in Met-T2D cases and mostly also significant in the South-Asian Surinamese. In line with the ASV analysis, no significantly altered pathways were present between TN-T2D cases and their matched controls in both ethnicities.

Figure 5B shows the most significantly altered pathways in Met-T2D cases compared to their controls for both ethnicities. Several pathways representing menaquinol biosynthesis, fermentation of pyruvate and TCA cycle IV had a higher abundance in Met-T2D cases in both ethnicities, whereas South-Asian Surinamese Met-T2D cases also displayed a significantly lower abundance of several amino acid biosynthesis pathways. Disentangling the effect of potential confounders revealed that African Surinamese Met-T2D cases still showed a significantly higher abundance of the selected pathways after adjusting for age, sex and BMI but most of the time not after additional correction for medication use (Supplementary Table S9). On the other hand, South-Asian Surinamese Met-T2D cases had significantly lower abundance for their top 11 most significant pathways after adjusting for both age, sex, BMI and medication use, except for the TCA cycle IV pathway, which was robustly present with a higher abundance in Met-T2D cases. These less abundant pathways were uniquely selected for South-Asian Surinamese, with significant interaction effects between Met-T2D and ethnicity. Subanalyses of models on the highlighted pathways above adjusted for age, sex and BMI in the groups with dietary information revealed either no significant effects for Met-T2D or the effect disappeared after adjusting for the dietary patterns.

Subsequent machine learning models could not distinguish T2D subjects from controls with a high accuracy based on the predicted pathways abundances for both African Surinamese datasets (average AUC Met-T2D cases vs. matched controls: 0.59 ± 0.06, average AUC TN-T2D cases vs. matched controls: 0.51 ± 0.06) and the South-Asian Surinamese TN-T2D cases vs. matched controls (average AUC 0.52 ± 0.12). Prediction performance of the model in the South-Asian Surinamese Met-T2D vs. matched controls was only modest, as indicated by the average AUC of 0.7 ± 0.06.

## 4. Discussion

In this study, we showed differences in gut microbiome α- and β–diversity measures, as well as in abundances of bacterial taxa (ASVs) and functional pathways between individuals with diabetes on metformin treatment and their healthy controls from two ethnic minority groups (South-Asian Surinamese and African Surinamese) living in the same geographical area and characterized by a high T2D prevalence. Furthermore, we showed that Met-T2D cases could be separated from their healthy controls based on their gut microbiome composition in both ethnicities using machine learning classifiers. Some of the Met-T2D-associated ASVs and pathways showed overlap between ethnicities. The gut microbiome of South-Asian Surinamese displayed more alterations between Met-T2D cases and controls, indicated by lower α-diversity measures and more significantly differentially abundant ASVs and pathways than the gut microbiome of the African Surinamese, when the same Met-T2D status was considered. By contrast, except for a lower richness and phylogenetic diversity in South-Asian Surinamese TN-T2D cases compared to their controls, we did not observe any other relation between TN-T2D and the gut microbiome in both ethnicities.

### 4.1. Gut Microbiota Composition Is Comparable in Treatment Naïve Diabetic Cases and Controls

Our statistically nonsignificant results in the ASV and pathway analyses for TN-T2D status in both ethnicities are in line with some previous studies [9,21]. However, several other studies have reported significant alterations in the gut microbiome of TN-T2D compared to controls [9,16,19,56]. Explanations could be either the use of shotgun sequencing data or a larger sample size. In this latter regard, our study included fewer TN-T2D cases than Met-T2D cases which could have resulted in a lower statistical power in the analysis. Furthermore, some of these previous studies did not use a matching strategy to account for potential confounders, whereas matching can greatly reduce the number of gut microbiome and T2D associations [57]. Taking all of this together suggests that true T2D patterns in treatment-naïve diabetes subjects might be subtle and complex and therefore requires high-resolution gut microbiome sequencing on large datasets [21]. In this regard, Falony et al. [58] estimated that a sample size of more than 1000 subjects is probably needed to adequately assess the relationship between obesity and microbiota composition in a cohort study, even when confounders such as age and gender are taken into account.

### 4.2. Overlapping Biomarkers between Ethnicities Were Previously Related to Metformin Use

Contrary to TN-T2D status, we did identify statistically significant results according to Met-T2D status. Those cases could express a more severe diabetic phenotype compared to the untreated diabetes cases. However, previous studies have reported that metformin use itself affects gut microbiome composition, even in healthy normoglycemic individuals [9,22,23,24,59,60]. Therefore, disentangling the effect of metformin from the effect of type 2 diabetes on the gut microbiome in our datasets is difficult. Interestingly, of all ASVs associated to Met-T2D in both ethnicities, almost all have been related to metformin use before in humans with the same direction. *Clostridium sensu stricto 1* [23] and *Peptostreptococcaceae Romboutsia* [24], *Terrisporobacter* [23], and especially *Intestinibacter bartlettii* [9,22,23,60] were decreased, while *Escherichia* [9,22,23,24] showed an increase after metformin treatment in previous studies. In our study, *Romboutsia* was identified as a robust and strong biomarker in the relation between Met-T2D and the gut microbiome in both ethnicities. Besides its potential relation to metformin use [24,61,62], *Romboutsia* was also previously negatively correlated with the abundance of *Enterobacteriaceae*, in line with the observed higher abundance of *Escherichia/Shigella* in Met-T2D cases in our study [63]. As for the other overlapping ASVs, the effect of Met-T2D remained significant on the abundance of *Clostridium sensu stricto 1*, *Peptostreptococcaceae Terrisporobacter* and *Intestinibacter bartlettii*, and *Escherichia/Shigella* after all adjustments in African Surinamese, while those significant effects vanished in South-Asian Surinamese after adjustment for other medication use (often not addressed in the previous studies). While interactions between Met-T2D and ethnicity were significant for *Peptostreptococcaceae Terrisporobacter* and *Intestinibacter bartlettii,* they were not for *Clostridium sensu stricto 1* and *Escherichia/Shigella*, indicating that associations in South-Asian Surinamese could not be excluded for these ASVs. By contrast, South-Asian Surinamese Met-T2D cases had a significantly higher abundance of *Lachnoclostridium*, also previously related to metformin use [21,23] which was not observed in African Surinamese and was robust against adjustment for confounders. Metformin use has also often been related to increased abundances of *Proteobacteria* abundance, *Akkermansia muciniphila,* and butyrate and propionate production potential. [9,18,22,23] Except for the association with *A. Muciniphila*, this was replicated in our study in both ethnicities, although represented by different ASVs and often only as important biomarkers in the machine learning analyses. The functional analysis revealed the same pattern. Almost all pathways that overlapped between both ethnicities have been related to metformin use before, including an increase in pathways corresponding to fermentation of pyruvate to butanoate or related to menaquinol (vitamin K_2_) biosynthesis and to TCA cycle. [22,64] However, the effect of Met-T2D on the abundance of these pathways was less robust against the adjustment for confounders in our study. Most of these ASVs and pathways were identified in randomized controlled trials in European descent populations. Herein, we showed that similar alterations were also observed in individuals from non-European descent although sometimes only in one ethnicity, which could suggest that metformin affects the gut microbiome in a universal way, while there might be some ethnic-specific effect on some taxa [18,21]. However, randomized controlled trials investigating the effect of metformin in other ethnicities are needed for confirmation. Furthermore, some of the abovementioned taxa were identified as altered in the same direction in treatment-naïve T2D subjects [10,19], including *Romboutsia* [10], *Intestinibacter bartlettii* [19] and *Lachnoclostridium* [10]; therefore, caution is needed before attributing those effects solely to metformin.

### 4.3. Biomarkers Related to Cardiometabolic Indicators Mostly Represented by Different ASVs across Ethnicities

Besides potential metformin-driven effects, we also observed patterns previously related to type 2 diabetes or other cardiometabolic parameters in our Met-T2D analyses. For instance, a lower abundance of butyrate producers has been reported in diabetes [9,10], which is consistent with our results of several ASVs assigned to potential butyrate producing genera that showed a lower abundance in Met-T2D cases in both ethnicities [18,65]. For both ethnicities, ASVs assigned to *Anaerostipes hadrus* were indeed significantly and robustly negatively associated with Met-T2D [66]. This species has been negatively correlated with BMI and blood glucose levels and exhibit a composite inositol catabolism-butyrate biosynthesis pathway that has been associated with lower metabolic disease risk [67]. Likewise, several ASVs assigned to *Lachnospiraceae*, a family containing several butyrate producers [65], were significantly and specifically less abundant in South-Asian Surinamese Met-T2D compared to their controls, with a strong interaction effect between Met-T2D and ethnicity. In addition, we identified ASVs previously related to blood pressure or to circulating lipid metabolites, including the *Clostridium sensu stricto 1* and *Peptostreptococcaceae* [68,69]. ASVs negatively associated with blood pressure, VLDL particles or small HDL particles in general were less abundant in Met-T2D cases compared to controls in our study, whereas ASVs positively associated with these features were more abundant. However, these associations were often not robust against the adjustment for confounders. These observations could illustrate the overall less healthy metabolic phenotype in individuals with T2D.

### 4.4. Alterations in Functional Potential of the Gut Microbiome Were More Frequent in South-Asian Surinamese

Finally, functional predictions revealed a large number of significantly different pathways in South-Asian Surinamese Met-T2D cases compared to controls, but only a small number in the African Surinamese, with significant interactions between Met-T2D and ethnicity for most pathways. This could relate to a more disturbed functional capacity of the gut microbiome but also to a better representation of South-Asian Surinamese taxa in the PICRUSt database. However, machine learning analyses did not reveal a clear distinction between Met-T2D cases and controls based on these pathways. South-Asian Surinamese Met-T2D cases in our study showed a reduced biosynthesis potential of several amino acids, including branched chain amino acids (BCAAs). Remarkably, BCAAs are often positively associated with insulin resistance or T2D, and BCAA synthesis was even enriched in samples after addition of metformin to an in vitro gut simulator [10,13,22]. Further research is therefore required if these pathways are specific for South-Asian Surinamese and if these represent a metformin-related or diabetes-related pattern.

### 4.5. Relation to Results in Other Cohorts with the Same Genetic Background

Previous sequence data of the gut microbiome in individuals with African and South-Asian genetic backgrounds have already revealed taxa associated with type 2 diabetes in those ethnic groups [16,17,21,61,70,71,72,73]. In Indian subjects, several studies have reported a higher abundance of *Escherichia* in treated T2D cases and a higher abundance of *Lactobacillus* in both treated and untreated T2D cases [16,17,73]. Taxa belonging to the *Lachnospiraceae* or *Ruminococcaceae* families were often enriched in controls compared to T2D cases. These results are in line with ours. By contrast, we did not identify *Prevotella* as a biomarker, and results regarding α-diversity were inconsistent across studies [16,17,73]. Recently, Alvarez-Silva et al. [21] analyzed the relation between the gut microbiome and T2D in a large Danish–Indian cohort (not living in the same geographic area) taking several potential confounders, including metformin and other medication use, into account. They could only discover metformin unrelated T2D-associated taxa in the total cohort but not in the Indian subcohort. In this Indian cohort, also no functional biomarkers remained significant after correction for all confounders. These results are in line with the results in our TN-T2D South-Asian Surinamese cases. Lastly, a higher abundance of *Lachnoclostridium,* but not *Escherichia/Shigella*, was observed in metformin-treated diabetes subjects compared to untreated ones in this Indian subpopulation [21]. While we did not compare treated and untreated diabetes subjects, these results are in line with our results in the metformin-treated South-Asian Surinamese cases after correction for all considered confounders. In Nigerian subjects, taxa from the families *Clostridiaceae* and *Peptostreptococcaceae* showed a lower abundance in T2D subjects (all or more than half on metformin treatment) in two studies compared to controls [70,71]. In addition, Doumatey et al. [70] mentioned a lower abundance of *Anaerostipes* in T2D subjects. These are all in agreement with our study. However, they reported a higher α-diversity in T2D subjects.

### 4.6. Strengths and Limitations

While several other studies have compared T2D subjects to controls from different ethnicities [8,9,21,25], the main strength of our study is the inclusion of two different ethnicities from the same geographic area. Both ethnicities were analyzed with the same univariate and machine learning pipelines, which allowed us to compare the results. Furthermore, we applied a matching procedure and stratified the diabetes subjects on metformin use to account for potential confounders. However, this study also has several limitations. First of all, while we reduced the effect of potential confounders as much as possible, we could not eliminate it completely. Biomarkers were only identified in the metformin treated diabetes subjects, but we were not able to disentangle the effect of metformin from a potential effect of diabetes stage. In some cases, the identified biomarkers were dependent on the model or transformation method used, indicating that some caution must be applied to interpret those results. Furthermore, statins, also known to affect the gut microbiome, were only taken by diabetes subjects, which could have masked some of the T2D effects [64,74]. Lastly, matching did not eliminate all differences in age, sex and BMI between healthy and diabetes subjects, especially for the South-Asian Surinamese Met-T2D vs. controls. This could also explain the less robust results for ASV abundance after adjusting for these confounders [57]. Another limitation of this study is the difference in sample size between the metformin-treated groups and the treatment-naïve groups, especially for the South-Asian Surinamese. In addition, diet information was only available for a small part of the samples, which resulted in different sample sizes and probably limited power in these analyses. By contrast, within treatment groups, samples sizes were comparable between both ethnicities. Therefore, comparisons across ethnicities were not limited by sample size. Furthermore, we used 16S rRNA sequencing data, which does not provide information at the species, strain or functional level, and we could have missed subtle changes. Moreover, functional prediction was performed using PICRUSt, which is limited to the included reference sequences and might not capture the true functional potential. For follow-up research, it would be interesting to analyze shotgun data in equally sized groups. Lastly, our study is cross-sectional, and therefore no causative statements of gut microbiome changes and type 2 diabetes or metformin use can be made. In the future, longitudinal data for both ethnicities could shed light on this causal aspect.

## 5. Conclusions

In conclusion, we examined ethnic specific type 2 diabetes-related alterations in the gut microbiome of two ethnic groups (South-Asian Surinamese and African Surinamese) living in the same geographical area. No statistically significant biomarkers were identified between controls and treatment-naïve subjects in both ethnicities. However, significant differences in gut microbiome composition and function were identified in metformin-treated T2D individuals compared to controls and more so in South-Asian Surinamese. The ASV with the strongest association was assigned to *Romboutsia* and was consistently less abundant in Met-T2D cases compared to controls in both ethnicities. Since this taxon was previously related to metformin use, as were some other identified markers, this may indicate a metformin-driven signal. Other identified taxa were previously related to diabetes, blood pressure or lipid profiles but were often represented by different ASVs in different ethnicities. Identifying metformin-unrelated, true type 2 diabetes patterns in the gut microbiome is thus complex. More research, preferably using shotgun sequencing at higher resolution in prospective studies, is necessary to investigate if different strains or species are indeed implicated in type 2 diabetes in both ethnicities to guide future prevention, diagnostic or therapeutic approaches.

## Figures and Tables

**Table 1:** Population characteristics. Overview of population characteristics for African Surinamese and South-Asian Surinamese for both metformin treated (Met-T2D) and treatment-naïve (TN-T2D) diabetes subjects and their matched controls.

	African Surinamese						South-Asian Surinamese					
	Met-T2D vs. Controls			TN-T2D vs. Controls			Met-T2D vs. Controls			TN-T2D vs. Controls		
	Healthy	T2D	*p*	Healthy	T2D	*p*	Healthy	T2D	*p*	Healthy	T2D	*p*
*n*	111	111		78	78		128	128		49	49	
Sex male	41 (36.9%)	38 (34.2%)	0.78 (*)	46 (59.0%)	41 (52.6%)	0.52 (*)	47 (36.7%)	75 (58.6%)	0.00073 (*)	22 (44.9%)	23 (46.9%)	1 (*)
Age (in years)	56.5 ± 6.8	58.7 ± 6.5	0.013 (+)	57 (53–61)	57 (52–62.75)	0.65 (&)	45 (33.85–51.3)	58 (54.8–64)	<2.2 × 10^−16^ (&)	53 (49–59)	58 (50–62)	0.047 (&)
BMI (in kg/m^2^)	29.2 ± 4.8	30.3 ± 4.8	0.087 (+)	28.95 ± 5.1	31.33 ± 6.5	0.012 (+)	24.1 (22.0–26.5)	27.7 (25.3–30.7)	2.0 × 10^−14^ (&)	25.8 (23.6–28.0)	26.5 (24.1–30.4)	0.11 (&)
*n* with diet information	33 (29.7%)	27 (24.3%)	0.45 (*)	24 (30.8%)	20 (25.6%)	0.59	44 (34.4%)	57 (44.5%)	0.12 (*)	15 (30.6%)	16 (32.7%)	1 (*)
PCDiet 1	−0.12 (−0.53–0.11)	−0.12 (−0.43–0.10)	0.79 (&)	−0.12 (−0.58–0.32)	−0.12 (−0.33–0.01)	0.90 (&)	−0.16 (−0.41–0.08)	−0.04 (−0.37–0.31)	0.16 (&)	−0.20 (−0.37− -0.05)	−0.24 (−0.45–0.19)	0.89 (&)
PCDiet 2	−0.44 (−0.72–0.06)	−0.36 (−0.74–0.03)	0.82 (&)	−0.42 (−0.73–0.14)	−0.30 (−0.77–0.23)	0.86 (&)	−0.11 (−0.52–0.48)	−0.34 (−0.63–0.38)	0.26 (&)	−0.39 (−0.69– -0.11)	−0.17 (−0.49–0.90)	0.14 (&)
Fasting glucose (mmol/L)	5.0 (4.8–5.3)	7.2 (6.4–8.4)	<2.2 × 10^−16^ (&)	5.1 (4.8–5.3)	7.0 (6.1–7.9)	<2.2 × 10^−16^ (&)	5 (4.8–5.2)	7.6 (6.4–8.6)	<2.2 × 10^−16^ (&)	5.1 (4.9–5.3)	6.9 (6–7.5)	1.2 × 10^−14^ (&)
HbA1c (mmol/mol)	36 (33–38)	51 (46–60)	<2.2 × 10^−16^ (&)	36 (32.3–37.8)	50 (48–53)	<2.2 × 10^−16^ (&)	36 (34–37)	53 (49–64)	<2.2 × 10^−16^ (&)	37 (35–38)	50 (48–53)	<2.2 × 10^−16^ (&)
MetSyn Yes	0 (0%)	104 (93.7%)	<2.2 × 10^−16^ ($)	0 (0%)	63 (80.8%)	<2.2 × 10^−16^ ($)	0 (0%)	123 (96.1%)	<2.2 × 10^−16^ ($)	0 (0%)	40 (81.6%)	<2.2 × 10^−16^ ($)
Central obesity Yes	86 (77.5%)	101 (91.0%)	0.0099(*)	51 (65.4%)	64 (82.1%)	0.029 (*)	77 (60.2%)	122 (95.3%)	3.8 × 10^−11^ (*)	38 (77.6%)	40 (81.6%)	0.80 (*)
High blood pressure Yes	80 (72.1%)	102 (91.9%)	0.00025 (*)	56 (71.2%)	67 (85.9%)	0.050 (*)	34 (26.6%)	116 (90.6%)	<2.2 × 10^−16^ (*)	21 (42.9%)	35 (71.4%)	0.0080 (*)
Low HDL Yes	2 (1.8%)	79 (71.2%)	<2.2 × 10^−16^ (*)	3 (3.8%)	39 (50%)	2.7 × 10^−10^ (*)	20 (15.6%)	107 (83.6%)	<2.2 × 10^−16^ (*)	4 (8.2%)	35 (71.4%)	6.0 × 10^−10^ (*)
High Triglycerides Yes	0 (0%)	71 (64.0%)	<2.2 × 10^−16^ ($)	0 (0%)	29 (37.2%)	1.5 × 10^−10^ ($)	7 (5.5%)	104 (81.3%)	<2.2 × 10^−16^ (*)	7 (14.3%)	25 (51.0%)	0.00025 (*)
High glucose Yes	0 (0%)	111 (100%)	<2.2 × 10^−16^ ($)	0 (0%)	73 (93.6%)	<2.2 × 10^−16^ ($)	0 (0%)	128 (100%)	<2.2 × 10^−16^ ($)	0 (0%)	42 (85.7%)	<2.2 × 10^−16^ ($)
Insulin use Yes	0 (0%)	25 (22.5%)	1.3 × 10^−8^ ($)	0 (0%)	0 (0%)	1 ($)	0 (0%)	25 (19.5%)	1.6 × 10^−8^ ($)	0 (0%)	0 (0%)	1 ($)
PPI use Yes	12 (10.8%)	22 (19.8%)	0.093 (*)	5 (6.4%)	12 (15.4%)	0.12 (*)	4 (3.1%)	34 (26.6%)	3.4 × 10^−7^ (*)	3 (6.1%)	6 (12.2%)	0.49 ($)
Statin use Yes	0 (0%)	59 (53.2%)	<2.2 × 10^−16^ ($)	0 (0%)	13 (16.7%)	0.00014 ($)	0 (0%)	90 (70.3%)	<2.2 × 10^−16^ ($)	0 (0%)	17 (34.7%)	2.8 × 10^−6^ ($)
Laxative use Yes	4 (3.6%)	0 (0%)	0.12 ($)	1 (1.3%)	1 (1.3%)	1 ($)	1 (0.8%)	4 (3.1%)	0.37 ($)	0 (0%)	4 (8.2%)	0.12 ($)
Beta blockers Yes	9 (8.1%)	27 (24.3%)	0.0020 (*)	7 (9.0%)	13 (16.7%)	0.23 (*)	3 (2.3%)	39 (30.5%)	3.5 × 10^−9^ (*)	2 (4.1%)	9 (18.4%)	0.055
Renin-angiotensin use Yes	21 (18.9%)	65 (58.6%)	3.1 × 10^−9^ (*)	14 (17.9%)	24 (30.8%)	0.093 (*)	7 (5.5%)	71 (55.5%)	<2.2 × 10^−16^ (*)	4 (8.2%)	12 (24.5%)	0.056

Differences in clinical characteristics are tested with either two sample *t*-test (+), Wilcoxon rank-sum test (&), Chi-squared test (*) or Fisher exact test ($).

## Data Availability

The HELIUS data are owned by the Amsterdam UMC, location AMC in Amsterdam, the Netherlands. Any researcher can request the data by submitting a proposal as outlined at http://www.heliusstudy.nl/.

## Supplementary Materials

The following are available online at https://www.mdpi.com/article/10.3390/nu13093289/s1, Table S1: Dietary patterns, Table S2: Alpha diversity, Table S3: Alpha diversity in diet cohort, Table S4: Beta diversity, Table S5: Beta diversity on diet subcohort, Table S6: Overview of the univariate Wilcoxon analysis for all ASVs, Table S7: Overview of significant associations between Met-T2D status and arcsin-square root transformed ASV abundance from linear regression models in African Surinamese and South-Asian Surinamese, Table S8: Overview of the univariate Wilcoxon analysis for all functional pathways, and Table S9: Overview of significant associations between Met-T2D status and log-transformed pathway abundance from linear regression models in the African Surinamese and South-Asian Surinamese.
