# Supplementary material for: Analyzing Type 2 Diabetes Associations with the Gut Microbiome in Individuals from Two Ethnic Backgrounds Living in the Same Geographic Area
Source: Nutrients. 2021 Sep 21;13(9):3289. doi: 10.3390/nu13093289 (PMC8468640; doi:10.3390/nu13093289)
Supplement: Supplementary file 1 [file nutrients-13-03289-s001.zip › Online-Only Supplemental Material.pdf]

Supplementary tables

Table S1: Dietary patterns

Overview of the factor loading of the different food groups for each dietary pattern. PC1 is considered as a “healthy” diet pattern, whereas PC2 represents a “Western” diet pattern. Significant factor loadings (≥ 0.3) are highlighted with yellow.

Table S2: Alpha diversity

Overview of associations between T2D status and alpha diversity for both ethnicities from the linear regression models. Met = metformin treated diabetes subjects vs matched controls, TN = treatment naïve diabetes subjects vs matched controls, Afr Surinamese = African Surinamese, SA Surinamese = South-Asian Surinamese, Combined = Afr Surinamese + SA Surinamese, Cov = covariate (age, sex, BMI) adjusted, cov + med = covariate and medication (age, sex, BMI, PPI, statins, beta blocker use and use of medication working on the renin-angiotensin system) adjusted, Coef = coefficient in linear model, T2D = Type 2 Diabetes, T2D:Ethnicity = interaction term between T2D and ethnicity.

Measure	Dataset	Model	Afr Surinamese						SA Surinamese						Combined
			F-stat total model	p-value total model	R^2	$AdjR^2$	Coef T2D	p-value T2D	F-stat total model	p-value total model	R^2	$AdjR^2$	Coef T2D	p-value T2D	p-value T2D:Ethnicity
Shannon	Met	Unadjusted	2.113	0.15	0.009512	0.00501	-0.08589	0.15	9.18	0.0027	0.03488	0.03108	-0.17324	0.0027	0.29
		Cov	1.078	0.37	0.01948	0.001406	-0.09598	0.12	2.553	0.040	0.03909	0.02378	-0.2331	0.0053	0.13
		Cov + med	0.5865	0.79	0.02155	-0.01519	-0.07044	0.38	1.357	0.22	0.04209	0.01107	-0.2270	0.030	0.15
	TN	Unadjusted	0.8629	0.35	0.005572	-0.0008851	-0.07337	0.35	1.869	0.17	0.0191	0.008882	-0.1190	0.18	0.71
		Cov	1.052	0.38	0.02711	0.001338	-0.09472	0.25	1.133	0.35	0.04645	0.005441	-0.1214	0.18	0.73
		Cov + med	0.5536	0.81	0.02925	-0.02358	-0.1038	0.24	1.534	0.16	0.1212	0.04221	-0.1930	0.05	0.58
Inverse Simpson	Met	Unadjusted	1.311	0.25	0.005922	0.001403	-1.969	0.25	5.78	0.017	0.02225	0.0184	-3.371	0.017	0.52
		Cov	0.5176	0.72	0.009452	-0.008807	-1.8368	0.30	1.469	0.21	0.02288	0.007311	-3.659	0.073	0.60
		Cov + med	0.5604	0.81	0.02061	-0.01617	-1.1248	0.63	1.08	0.38	0.03379	0.002492	-3.005	0.24	0.68
	TN	Unadjusted	0.4605	0.50	0.002981	-0.003493	-1.636	0.50	0.4709	0.49	0.004881	-0.005485	-1.612	0.50	0.99
		Cov	0.6582	0.62	0.01714	-0.008898	-2.002	0.42	0.3449	0.85	0.01462	-0.02776	-1.167	0.64	0.99
		Cov + med	0.3643	0.94	0.01944	-0.03392	-2.027	0.45	0.5973	0.78	0.05095	-0.03436	-2.724	0.32	0.93
Richness	Met	Unadjusted	2.082	0.15	0.009375	0.004872	-28.72	0.15	11.73	0.00072	0.04415	0.04039	-69.48	0.00072	0.16
		Cov	0.8282	0.51	0.01504	-0.003119	-33.54	0.10	3.36	0.011	0.05082	0.0357	-87.89	0.0030	0.073
		Cov + med	0.655	0.73	0.02401	-0.01265	-18.68	0.48	1.789	0.080	0.05476	0.02415	-80.82	0.029	0.088
	TN	Unadjusted	0.4306	0.51	0.002788	-0.003687	-15.64	0.51	2.135	0.15	0.02175	0.01156	-46.47	0.15	0.43
		Cov	0.9446	0.44	0.02441	-0.001432	-16.30	0.51	2.705	0.04	0.1042	0.06569	-49.89	0.12	0.46
		Cov + med	0.5441	0.82	0.02876	-0.0241	-13.81	0.60	3.659	0.0010	0.2475	0.1799	-78.67	0.021	0.31
Faith's PD	Met	Unadjusted	4.637	0.032	0.02064	0.01619	-1.926	0.032	6.682	0.010	0.02563	0.0218	-2.149	0.010	0.86
		Cov	1.779	0.13	0.03175	0.0139	-2.200	0.018	1.753	0.14	0.02718	0.01168	-2.597	0.032	0.51
		Cov + med	1.006	0.43	0.0364	0.0002087	-1.716	0.15	1.124	0.35	0.03513	0.003882	-2.052	0.18	0.59
	TN	Unadjusted	0.04198	0.84	0.0002726	-0.006219	0.2106	0.84	0.6556	0.42	0.006782	-0.003564	-1.016	0.42	0.45
		Cov	1.813	0.13	0.04582	0.02054	0.01585	0.99	1.95	0.11	0.07738	0.0377	-1.320	0.30	0.44
		Cov + med	1.325	0.24	0.06727	0.01651	0.06379	0.95	3.076	0.0042	0.2166	0.1462	-2.740	0.043	0.26

Table S3: Alpha diversity in diet cohort

Overview of associations between T2D status and alpha diversity for both ethnicities from the linear regression models in the subsample with available diet information. Met = metformin treated diabetes subjects vs matched controls, TN = treatment naïve diabetes subjects vs matched controls, Afr Surinamese = African Surinamese, SA Surinamese = South-Asian Surinamese, Combined = Afr Surinamese + SA Surinamese, Diet = diet adjusted (PCDiet1 and PCDiet2), Cov = covariate (age, sex, BMI) adjusted, cov + diet = covariate and diet adjusted (age, sex, BMI, PCDiet1, PCDiet2), cov + med = covariate and medication (age, sex, BMI, PPI, statins, beta blocker use and use of medication working on the renin-angiotensin system) adjusted, cov + med + diet = covariate, medication and diet adjusted (age, sex, BMI, PPI, statins, beta blocker use, use of medication working on the renin-angiotensin system, PCDiet1 and PCDiet2), Coef = coefficient in linear model, T2D = Type 2 Diabetes, T2D:Ethnicity = interaction term between T2D and ethnicity.

Measure	Dataset	Model	Afr Surinamese						SA Surinamese						Combined
			**F-stat total model**	**p-value total model**	**R^2**	**AdjR^2**	**Coef T2D**	**p-value T2D**	**F-stat total model**	**p-value total model**	**R^2**	**AdjR^2**	**Coef T2D**	**p-value T2D**	**p-value T2D:Ethnicity**
Shannon	Met	Unadjusted	0.4579	0.50	0.007833	-0.00927	-0.08125	0.50	0.5711	0.45	0.005736	-0.0043	-0.06674	0.45	0.92
		Diet	0.3904	0.76	0.02048	-0.03199	-0.07580	0.54	2.875	0.040	0.08167	0.05327	-0.1268	0.16	0.81
		Cov	1.014	0.41	0.0687	0.000972	-0.07748	0.52	0.4666	0.76	0.01907	-0.0218	-0.08530	0.54	0.81
		Cov + diet	0.8125	0.57	0.08424	-0.01943	-0.07780	0.53	1.769	0.11	0.1015	0.0441	-0.08722	0.52	0.82
		Cov + med	0.6882	0.70	0.09744	-0.04414	-0.00131	0.99	0.5871	0.79	0.04857	-0.03416	-0.1356	0.40	0.90
		Cov + med + diet	0.6399	0.77	0.1155	-0.065	0.006712	0.97	1.506	0.15	0.1433	0.04816	-0.1641	0.30	0.95
	TN	Unadjusted	0.6259	0.43	0.01468	-0.00877	0.1141	0.43	0.02525	0.87	0.0008698	-0.03358	0.02727	0.87	0.70
		Diet	1.762	0.17	0.1167	0.05051	0.1306	0.36	0.9585	0.43	0.09625	-0.00416	0.08904	0.62	0.83
		Cov	0.3165	0.87	0.03144	-0.0679	0.1404	0.37	0.7467	0.57	0.103	-0.03495	-0.02894	0.88	0.64
		Cov + diet	1.038	0.42	0.144	0.005232	0.1634	0.28	1.018	0.44	0.2029	0.003573	-0.009171	0.96	0.73
		Cov + med	0.532	0.82	0.1084	-0.09537	0.1394	0.41	1.115	0.39	0.2886	0.02987	-0.1722	0.40	0.66
		Cov + med + diet	0.9328	0.52	0.2204	-0.01588	0.1895	0.26	1.496	0.21	0.4279	0.1419	-0.3287	0.16	0.75
Inverse Simpson	Met	Unadjusted	0.5157	0.48	0.008813	-0.00827	-2.309	0.48	0.6256	0.43	0.006279	-0.00375	-1.696	0.43	0.87
		Diet	0.5263	0.67	0.02742	-0.02468	-2.039	0.53	4.566	0.0049	0.1237	0.09664	-3.684	0.084	0.79
		Cov	1.057	0.39	0.07141	0.003875	-2.335	0.47	0.7418	0.57	0.02998	-0.01044	-3.273	0.33	0.89
		Cov + diet	0.8251	0.56	0.08543	-0.01811	-2.216	0.50	3.008	0.0098	0.1611	0.1075	-3.729	0.24	0.99
		Cov + med	0.93	0.50	0.1273	-0.00957	0.3296	0.94	0.8973	0.52	0.07237	-0.008288	-3.822	0.32	0.98
		Cov + med + diet	0.8578	0.58	0.149	-0.02469	0.7191	0.87	2.316	0.018	0.2047	0.1163	-5.247	0.16	0.86
	TN	Unadjusted	0.3884	0.54	0.009163	-0.01443	3.240	0.54	0.08095	0.78	0.002784	-0.0316	-1.315	0.78	0.54
		Diet	1.56	0.21	0.1047	0.03757	3.953	0.44	1.654	0.20	0.1553	0.06141	0.8533	0.86	0.64
		Cov	0.2573	0.90	0.02571	-0.07422	4.350	0.44	0.5667	0.69	0.08019	-0.06132	-3.008	0.56	0.44
		Cov + diet	0.9217	0.49	0.13	-0.01105	5.308	0.33	1.27	0.31	0.241	0.05122	-0.5832	0.91	0.53
		Cov + med	0.585	0.78	0.1179	-0.08366	4.142	0.50	0.8662	0.56	0.2395	-0.03699	-5.853	0.31	0.60
		Cov + med + diet	0.9258	0.52	0.2191	-0.01755	5.996	0.32	1.189	0.35	0.3728	0.05926	-5.967	0.36	0.71

Richness	Met	Unadjusted	0.2307	0.63	0.003962	-0.01321	-17.73	0.63	0.3305	0.57	0.003327	-0.00674	-16.76	0.57	0.98
		Diet	0.2478	0.86	0.0131	-0.03977	-17.52	0.64	3.68	0.015	0.1022	0.07441	-33.46	0.25	0.79
		Cov	0.1211	0.97	0.008731	-0.06336	-17.55	0.65	0.7159	0.58	0.02897	-0.01149	-16.06	0.72	0.90
		Cov + diet	0.1712	0.98	0.01902	-0.09204	-17.65	0.65	1.922	0.085	0.1093	0.05244	-9.235	0.83	0.85
		Cov + med	0.2361	0.98	0.03571	-0.1156	23.25	0.66	0.6973	0.69	0.05717	-0.02482	-28.97	0.58	0.97
		Cov + med + diet	0.2568	0.99	0.0498	-0.1441	25.41	0.64	1.488	0.16	0.1419	0.04657	-23.07	0.66	0.96
	TN	Unadjusted	0.6362	0.43	0.01492	-0.00853	28.53	0.43	1.252	0.27	0.04137	0.008314	62.96	0.27	0.59
		Diet	0.8389	0.48	0.0592	-0.01136	32.61	0.37	1.057	0.38	0.1051	0.005679	86.04	0.16	0.50
		Cov	0.8784	0.49	0.08265	-0.01144	25.94	0.49	1.248	0.32	0.161	0.03197	51.99	0.40	0.47
		Cov + diet	1.033	0.42	0.1434	0.004545	31.91	0.39	1.028	0.43	0.2044	0.005495	53.55	0.44	0.49
		Cov + med	0.981	0.47	0.1832	-0.00355	25.31	0.53	1.358	0.27	0.3306	0.0872	2.586	0.97	0.57
		Cov + med + diet	1.067	0.41	0.2443	0.01527	35.82	0.38	1.425	0.24	0.416	0.124	-49.70	0.52	0.59
Faith's PD	Met	Unadjusted	0.7491	0.39	0.01275	-0.00427	-1.422	0.39	0.4897	0.49	0.004922	-0.00512	-0.8776	0.49	0.79
		Diet	0.6851	0.56	0.0354	-0.01627	-1.506	0.37	1.382	0.25	0.04099	0.01133	-1.282	0.32	0.91
		Cov	0.8851	0.48	0.06048	-0.007854	-1.375	0.41	0.1225	0.97	0.005077	-0.03638	-0.9774	0.62	0.64
		Cov + diet	0.9908	0.44	0.1009	-0.00093	-1.525	0.36	0.855	0.53	0.05175	-0.008774	-0.7705	0.69	0.55
		Cov + med	0.5994	0.77	0.08595	-0.05743	-0.08722	0.97	0.4001	0.92	0.03362	-0.05041	-1.765	0.44	0.71
		Cov + med + diet	0.6965	0.72	0.1245	-0.05423	-0.1773	0.94	0.8445	0.59	0.08578	-0.0158	-0.1723	0.46	0.64
	TN	Unadjusted	0.4957	0.49	0.01166	-0.01187	1.456	0.49	1.387	0.25	0.04564	0.01273	2.78	0.25	0.68
		Diet	0.8961	0.45	0.06298	-0.0073	1.418	0.50	1.381	0.27	0.1331	0.03674	3.939	0.12	0.48
		Cov	0.6226	0.65	0.06002	-0.03639	1.649	0.45	1.651	0.19	0.2026	0.07988	1.104	0.66	0.79
		Cov + diet	0.8023	0.57	0.1151	-0.02837	1.735	0.43	1.342	0.28	0.2512	0.06402	1.471	0.60	0.66
		Cov + med	0.6444	0.74	0.1284	-0.07084	1.528	0.53	1.311	0.29	0.3228	0.07656	-1.051	0.71	0.88
		Cov + med + diet	0.7107	0.71	0.1772	-0.07214	1.834	0.45	1.566	0.19	0.4392	0.1587	-2.932	0.36	0.75

Table S4: Beta diversity

Overview of associations between T2D status and beta diversity for both ethnicities from PERMANOVA. Met = metformin treated diabetes subjects vs matched controls, TN = treatment naïve diabetes subjects vs matched controls, Afr Surinamese = African Surinamese, SA Surinamese = South-Asian Surinamese, Combined = Afr Surinamese + SA Surinamese, Cov = covariate (age, sex, BMI) adjusted, cov + med = covariate and medication (age, sex, BMI, PPI, statin, beta blocker use, use of medication working on the renin-angiotensin system) adjusted, T2D = Type 2 Diabetes, T2D:Ethnicity = interaction term between T2D and ethnicity.

Measure	Dataset	Model	Afr Surinamese						SA Surinamese						Combined
			**DF**	**SumsOfSqs**	**MeanSqs**	**Fmodel**	**R^2 T2D**	**p-value T2D**	**DF**	**SumsOfSqs**	**MeanSqs**	**Fmodel**	**R^2 T2D**	**p-value T2D**	**p-value T2D:Ethnicity**
Bray-Curtis	Met	Unadjusted	1	0.400	0.4000	1.520	0.00686	0.033	1	0.850	0.8503	3.233	0.01257	0.0002	0.68
		Cov	1	0.418	0.4183	1.598	0.00718	0.025	1	0.381	0.3809	1.455	0.00563	0.061	0.74
		Cov + med	1	0.406	0.4063	1.555	0.00697	0.029	1	0.319	0.3186	1.220	0.00471	0.17	0.75
	TN	Unadjusted	1	0.295	0.2954	1.157	0.00746	0.22	1	0.345	0.345	1.363	0.014	0.084	0.61
		Cov	1	0.328	0.3282	1.294	0.00829	0.13	1	0.2906	0.2906	1.156	0.01179	0.22	0.62
		Cov + med	1	0.292	0.2920	1.154	0.00737	0.23	1	0.2618	0.2618	1.049	0.01062	0.35	0.61
Weighted UniFrac	Met	Unadjusted	1	0.0991	0.09908	1.043	0.00472	0.34	1	0.5335	0.5335	5.291	0.02041	0.0013	0.17
		Cov	1	0.0988	0.09883	1.049	0.00471	0.34	1	0.2493	0.2493	2.500	0.00954	0.033	0.39
		Cov + med	1	0.1263	0.1263	1.344	0.00601	0.19	1	0.2010	0.2011	2.023	0.00769	0.071	0.40
	TN	Unadjusted	1	0.1113	0.1113	1.111	0.00716	0.29	1	0.1203	0.1203	1.236	0.01271	0.24	0.77
		Cov	1	0.1442	0.1442	1.460	0.00928	0.18	1	0.0769	0.07685	0.7990	0.00812	0.54	0.78
		Cov + med	1	0.1037	0.1037	1.051	0.00667	0.33	1	0.0878	0.08782	0.9099	0.00928	0.44	0.80
Unweighted UniFrac	Met	Unadjusted	1	0.258	0.2584	1.610	0.00727	0.019	1	0.440	0.4398	2.668	0.0104	0.0005	0.85
		Cov	1	0.271	0.2707	1.691	0.00761	0.017	1	0.275	0.2747	1.671	0.00649	0.020	0.81
		Cov + med	1	0.194	0.1936	1.209	0.00544	0.13	1	0.206	0.2063	1.257	0.00488	0.11	0.82
	TN	Unadjusted	1	0.1275	0.1275	0.8237	0.00532	0.83	1	0.1638	0.1639	1.009	0.0104	0.39	0.69
		Cov	1	0.1417	0.1417	0.9224	0.00591	0.58	1	0.1703	0.1703	1.058	0.01081	0.30	0.64
		Cov + med	1	0.1442	0.1442	0.9383	0.00602	0.53	1	0.1721	0.1721	1.083	0.01092	0.26	0.62

Table S5: Beta diversity on diet sub cohort

Overview of associations between T2D status and beta diversity for both ethnicities from PERMANOVA analyses in the subsample with available diet information. Met = metformin treated diabetes subjects vs matched controls, TN = treatment naïve diabetes subjects vs matched controls, Afr Surinamese = African Surinamese, SA Surinamese = South-Asian Surinamese, Combined = Afr Surinamese + SA Surinamese, Diet = diet adjusted (PCDiet1 and PCDiet2), Cov = covariate (age, sex, BMI) adjusted, cov + diet = covariate and diet adjusted (age, sex, BMI, PCDiet1 and PCDiet2), cov + med = covariate and medication adjusted (age, sex, BMI, PPI, statin, beta blocker use, use of medication working on the renin-angiotensin system), cov + med + diet = covariate, medication and diet adjusted (age, sex, BMI, PPI, statin, beta blocker use, use of medication working on the renin-angiotensin system, PCDiet1 and PCDiet2), T2D = Type 2 Diabetes, T2D:Ethnicity = interaction term between T2D and ethnicity.

Measure	Dataset	Model	Afr Surinamese						SA Surinamese						Combined
			**DF**	**SumsOfSqs**	**MeanSqs**	**Fmodel**	**R^2 T2D**	**p-value T2D**	**DF**	**SumsOfSqs**	**MeanSqs**	**Fmodel**	**R^2 T2D**	**p-value T2D**	**p-value T2D:Ethnicity**
Bray-Curtis	Met	Unadjusted	1	0.2709	0.2709	1.033	0.0175	0.37	1	0.3796	0.3796	1.405	0.01399	0.081	0.66
		Diet	1	0.2706	0.2706	1.031	0.01748	0.37	1	0.3297	0.3297	1.235	0.01215	0.17	0.64
		Cov	1	0.2773	0.2773	1.050	0.01791	0.35	1	0.2141	0.2141	0.7943	0.00789	0.77	0.65
		Cov + diet	1	0.2763	0.2764	1.046	0.01785	0.35	1	0.2195	0.2195	0.8217	0.00809	0.74	0.66
		Cov + med	1	0.2483	0.2483	0.9333	0.01604	0.55	1	0.2654	0.2654	0.9985	0.00978	0.42	0.62
		Cov + med + diet	1	0.2528	0.2528	0.9474	0.01633	0.52	1	0.2924	0.2924	1.109	0.01078	0.28	0.58
	TN	Unadjusted	1	0.2349	0.2349	0.8854	0.02065	0.59	1	0.4124	0.4124	1.674	0.05456	0.045	0.096
		Diet	1	0.2353	0.2353	0.8918	0.02068	0.59	1	0.393	0.3930	1.571	0.052	0.060	0.067
		Cov	1	0.2521	0.2521	0.9361	0.02216	0.51	1	0.3238	0.3238	1.356	0.04284	0.12	0.10
		Cov + diet	1	0.2528	0.2528	0.9422	0.02222	0.49	1	0.313	0.3130	1.293	0.04142	0.16	0.069
		Cov + med	1	0.2390	0.2390	0.8773	0.02101	0.61	1	0.2529	0.2529	1.109	0.03347	0.29	0.21
		Cov + med + diet	1	0.2388	0.2388	0.8794	0.02099	0.61	1	0.2576	0.2576	1.145	0.03408	0.25	0.14
Weighted UniFrac	Met	Unadjusted	1	0.0704	0.07040	0.719	0.01224	0.62	1	0.1524	0.1524	1.486	0.01478	0.16	0.67
		Diet	1	0.0673	0.06729	0.6811	0.0117	0.66	1	0.1710	0.1710	1.728	0.01659	0.11	0.71
		Cov	1	0.0798	0.07982	0.8052	0.01388	0.54	1	0.0532	0.05317	0.5263	0.00516	0.80	0.69
		Cov + diet	1	0.0771	0.07708	0.7693	0.01341	0.58	1	0.0602	0.06021	0.6101	0.00584	0.72	0.74
		Cov + med	1	0.0821	0.08206	0.8364	0.01427	0.51	1	0.0725	0.07247	0.7284	0.00703	0.59	0.63
		Cov + med + diet	1	0.087	0.08704	0.8770	0.01514	0.47	1	0.0961	0.09608	0.9874	0.00932	0.38	0.66
	TN	Unadjusted	1	0.0612	0.06118	0.6018	0.01413	0.72	1	0.1659	0.1659	1.659	0.05412	0.14	0.13
		Diet	1	0.0655	0.06554	0.6386	0.01513	0.70	1	0.1176	0.1176	1.1525	0.03836	0.30	0.14
		Cov	1	0.0676	0.06758	0.6426	0.01561	0.69	1	0.09916	0.09916	1.085	0.03234	0.32	0.14
		Cov + diet	1	0.0713	0.07130	0.6682	0.01646	0.68	1	0.07087	0.07087	0.7558	0.02311	0.55	0.12
		Cov + med	1	0.0436	0.04358	0.4141	0.01006	0.91	1	0.1091	0.1091	1.255	0.03559	0.26	0.28
		Cov + med + diet	1	0.0493	0.04928	0.4618	0.01138	0.87	1	0.08245	0.08245	0.9503	0.02689	0.40	0.25
Unweighted UniFrac	Met	Unadjusted	1	0.1520	0.1520	0.9670	0.0164	0.47	1	0.1803	0.1803	1.096	0.01095	0.24	0.91
		Diet	1	0.1548	0.1548	0.9860	0.01671	0.42	1	0.1805	0.1805	1.098	0.01097	0.25	0.87
		Cov	1	0.1499	0.1499	0.9529	0.01617	0.49	1	0.1366	0.1366	0.8308	0.0083	0.80	0.89

		Cov + diet	1	0.1547	0.1547	0.9880	0.01669	0.41	1	0.1368	0.1368	0.8341	0.00831	0.80	0.81
		Cov + med	1	0.1165	0.1165	0.7324	0.01257	0.96	1	0.1541	0.1541	0.9477	0.00936	0.51	0.84
		Cov + med + diet	1	0.1186	0.1186	0.7488	0.0128	0.95	1	0.1601	0.1601	0.9880	0.00973	0.42	0.73
	TN	Unadjusted	1	0.1215	0.1215	0.7586	0.01774	0.90	1	0.2049	0.2049	1.274	0.04209	0.10	0.46
		Diet	1	0.1213	0.1213	0.7627	0.01771	0.89	1	0.2198	0.2198	1.351	0.04515	0.074	0.26
		Cov	1	0.1314	0.1314	0.8266	0.01918	0.75	1	0.1654	0.1654	1.062	0.03398	0.29	0.46
		Cov + diet	1	0.132	0.1320	0.8367	0.01928	0.72	1	0.1561	0.1561	0.9877	0.03206	0.41	0.31
		Cov + med	1	0.1251	0.1251	0.7841	0.01827	0.85	1	0.1343	0.1343	0.8706	0.02758	0.69	0.57
		Cov + med + diet	1	0.1288	0.1288	0.8134	0.0188	0.78	1	0.1446	0.1446	0.9394	0.02970	0.52	0.42

Supplementary Table S6

Overview of the univariate Wilcoxon analysis for all ASVs. Results are provided for the Wilcoxon statistic itself, the unadjusted p-value and the adjusted p-value after correction for multiple testing based on the Benjamini-Hochberg correction. ASVs are provided as (ASVname)_(taxonmy_name). ASVs not included for in analyses after filtering are indicated with NaN. Afr Sur Met-T2D = African Surinamese metformin treated T2D subjects and matched controls, Afr Sur TN-T2D = African Surinamese treatment naïve T2D subjects and matched controls, SA Sur Met-T2D = South-Asian Surinamese metformin treated T2D subjects and matched controls, SA Sur TN-T2D = South-Asian Surinamese treatment naïve T2D subjects and matched controls.

Supplementary Table S7

Overview of significant associations between Met-T2D status and arcsin-square root transformed ASV abundance from linear regression models in African Surinamese and South-Asian Surinamese. Results are provided as regression coefficient and p-value for T2D in the unadjusted, covariate adjusted (age, sex and BMI) and covariate and medication adjusted (age, sex, BMI and use of PPI, statins, beta blockers or medication working on the renin-angiotensin system) models for both ethnicities. The p-value for the interaction between Met-T2D and ethnicity as well as the results for the Wilcoxon analysis (adjusted for multiple testing) are also provided. Results are also shown for the diet subcohort on the same models and with additional adjustment for dietary patterns.

Supplementary Table S8

Overview of the univariate Wilcoxon analysis for all functional pathways. Results are provided for the Wilcoxon statistic itself, the unadjusted p-value and the adjusted p-value after correction for multiple testing based on the Benjamini-Hochberg correction. Pathways

not included for the analysis after filtering are indicated with NaN. Afr Sur Met-T2D = African Surinamese metformin treated T2D subjects and matched controls, Afr Sur TN-T2D = African Surinamese treatment naïve T2D subjects and matched controls, SA Sur Met-T2D = South-Asian Surinamese metformin treated T2D subjects and matched controls, SA Sur TN-T2D = South-Asian Surinamese treatment naïve T2D subjects and matched controls.

Supplementary Table S9

Overview of significant associations between Met-T2D status and log transformed pathway abundance from linear regression models in the African Surinamese and South-Asian Surinamese. Results are provided as regression coefficient and p-value for T2D in the unadjusted, covariate adjusted (age, sex and BMI) and covariate and medication adjusted (age, sex, BMI and use of PPI, statins, beta blockers or medication working on the renin-angiotensin system) models for both ethnicities. The p-value for the interaction term between Met-T2D and ethnicity as well as the results for the Wilcoxon analysis (adjusted for multiple testing) are also provided. Results are also shown for the diet subcohort on the same models and with additional adjustment for dietary patterns.
